# Assessing the Role of Statins as an Adjunctive Anti-VEGF Therapy for Clinically Significant Macular Edema (CSME) in Type 2 Diabetes Mellitus

**DOI:** 10.22336/rjo.2025.80

**Published:** 2025

**Authors:** Ashish Markan, Aniruddha Agarwal, Deeksha Katoch, Sanjay Bhadadaxys, Vishali Gupta, Reema Bansal

**Affiliations:** 1 Eye7 Chaudhary Eye Centre, New Delhi, India; 2 Cleveland Clinic, Abu Dhabi, United Arab Emirates; 3 Department of Ophthalmology, Post Graduate Institute of Medical Education and Research, Chandigarh, India; 4 Department of Endocrinology, Post Graduate Institute of Medical Education and Research, Chandigarh, India

**Keywords:** statin therapy, CSME, diabetic retinopathy, anti-VEGF, adjunct therapy, type 2 diabetes, BCVA = Best Corrected Visual Acuity, CAD = Coronary Artery Disease, CKD = Chronic Kidney Disease, CMT = Central Macular Thickness, CSME = Clinically Significant Macular Edema, COVID = Coronavirus Disease, DME = Diabetic Macular Edema, DM = Diabetes Mellitus, ETDRS = Early Treatment Diabetic Retinopathy Study, FBS = Fating Blood Sugar, FFA = Fundus Fluorescein Angiography, HDL = High Density Lipoprotein, HTN = Hypertension, IOP = Intraocular Pressure, KFT = Kidney Function Tests, LDL = Low Density Lipoprotein, LFT = Liver Function Tests, NPDR = Non-Proliferative Diabetic Retinopathy, OCT = Optical Coherence Tomography, PRN = Pro Re Nata, PTH = Para Thyroid Hormone, SGOT = Serum Glutamic Oxaloacetic Transaminase, SGPT = Serum Glutamic Pyruvic Transaminase, SPSS = Statistical Package for Social Sciences, TG = Triglycerides, VEGF - Vascular Endothelial Growth Factor

## Abstract

**Aim:**

This study aimed to evaluate the effectiveness of statin therapy as an adjunctive treatment to anti-VEGF therapy in type 2 diabetic patients with non-proliferative diabetic retinopathy (NPDR) and clinically significant macular edema (CSME).

**Materials and methods:**

In this prospective, randomized interventional study, patients were randomized into two groups: Group A received low-dose atorvastatin (10-20 mg), and Group B received high-dose atorvastatin (30-40 mg). All participants also received three loading doses of intravitreal ranibizumab (0.5 mg) at monthly intervals, followed by pro re nata treatment over six months. Primary outcomes included the number of anti-VEGF injections required, best-corrected visual acuity (BCVA), and central macular thickness (CMT). Serum VEGF levels were measured at baseline and six months.

**Results:**

The mean number of injections over six months was 3.4, with no significant difference between Group A (3.55) and Group B (3.33) (p = 0.24). Group A demonstrated substantial improvement in BCVA at both 3 and 6 months, accompanied by a notable reduction in CMT. In contrast, Group B’s BCVA improvement was only significant at 3 months, with less consistent CMT reduction at 6 months. Serum VEGF levels decreased in Group A but increased in Group B, though these changes were not statistically significant.

**Discussion:**

The findings suggest that low-dose atorvastatin, when used in conjunction with anti-VEGF therapy, may provide superior functional and anatomical outcomes in patients with CSME compared to high-dose statin therapy. The observed reduction in central macular thickness and improvement in visual acuity indicate a potential adjunctive benefit of statins, likely due to their pleiotropic effects, including anti-inflammatory and anti-angiogenic properties. Although the number of injections required was similar between groups, the better response in the low-dose group highlights the need for further investigation into the dose-dependent effects of statins in the management of diabetic macular edema.

**Conclusions:**

Low-dose atorvastatin (10-20 mg) as an adjunct to anti-VEGF therapy resulted in better functional and anatomical outcomes in diabetic patients with CSME compared to high-dose atorvastatin. These findings suggest potential additional benefits of low-dose statins in managing patients with chronic subdural hematoma (CSME).

## Introduction

Diabetes mellitus (DM) is a common chronic systemic disorder that may lead to sight-threatening complications such as diabetic macular edema (DME), proliferative diabetic retinopathy (PDR), retinal artery/vein occlusions, and tractional or combined retinal detachment [**1**]. One out of three people with diabetes suffers from Diabetic Retinopathy, with reported rates of DME reaching 7% in these patients [**2-6**]. DME is the leading cause of visual loss and legal blindness in people with diabetes. Vascular endothelial growth factor (VEGF) is considered a major mediator in the pathogenesis of diabetic macular edema (DME) [**7,8**]. VEGF upregulates angiogenesis and disrupts the blood-retinal barrier (BRB) by damaging the tight junctions between retinal endothelial cells. Other comorbidities implicated in the development of DME include chronic hyperglycemia, hypertension, and hyperlipidemia [**9**]. Several treatment modalities have been developed to address the condition, each with its advantages and disadvantages. Regardless of the therapy chosen, controlling other systemic comorbidities is also crucial in improving treatment outcomes [**10**].

Dyslipidemia seems to have a direct role in the development of clinically significant macular edema (CSME) [**11,12**]. Lowering of serum lipids has been shown to benefit both diabetic retinopathy and maculopathy. Some lipid-lowering agents, such as fibrates and statins, have been extensively studied in diabetic patients with retinopathy and have proven effective [**13-15**].

Statins exert lipid-lowering and lipid-nondependent effects. The lipid-independent effects of statins, referred to as pleiotropic, include improving or restoring endothelial function, decreasing oxidative stress, and exhibiting antithrombotic and immunomodulatory activity [**16**].

The anti-angiogenic action of statins has been extensively studied both clinically and experimentally. The dose-dependent action of statins on angiogenesis is confusing. Studies have shown that statins exert a biphasic effect: pro-angiogenic at low doses and anti-angiogenic at high doses [**17,18**]. In contrast, various clinical and experimental studies have demonstrated the anti-angiogenic action of statins, even at low doses [**19-21**]. Studies suggest a significant reduction in plasma VEGF concentrations with statin therapy [**19,22**]. This effect depends on the duration of treatment, low-density lipoproteins (LDL)-lowering activity, lipophilicity of statins, and health status of the studied individuals [**22**]. Studies have shown that statins exert an anti-VEGF action with prolonged use, without causing any severe adverse events. There is consolidated evidence in the literature that statins, when administered in high doses, have additional benefits. This is leading to increased statin use and the use of more intensive regimens [**23,24**]. Hence, the safety of this group of drugs is of considerable importance. Administering statins at high doses, up to 80 mg, is safe and well-tolerated [**24**]. The only well-documented, consistent adverse effects associated with statins are muscle toxicity, including myopathy and rhabdomyolysis, and effects on liver enzymes.

The drug is widely used for its systemic benefits, and its effects on the retina have been demonstrated by its ability to resolve hard exudates in CSME. However, its direct anti-angiogenic effect on the retina is being recently evaluated [**21,22**]. This prospective randomized interventional study was conducted to assess the effectiveness of statin therapy as an adjunctive anti-VEGF therapy in the management of type 2 diabetic patients with NPDR with CSME.

## Materials and methods

This was a prospective, interventional randomized study of diabetic patients with NPDR and CSME visiting the retina clinic of the Advanced Eye Centre, PGIMER, Chandigarh. The study was registered under Clinical Trials Registry-India (REF/2020/06/034527). The study was conducted in accordance with the Principles of the Helsinki Declaration. Ethical clearance was obtained from the Institutional Review Board.

Patients (age ≥18 years) with NPDR and CSME, who were treatment naïve (not having received any form of treatment for CSME in the form of anti-VEGF, intravitreal steroids, or laser therapy) and were willing to give consent and come for regular follow-up, were included in the study. CSME was defined as per ETDRS guidelines [**25**]. Additionally, patients who were not treatment-naïve but had received any of the above-mentioned therapies for CSME at least 3 months before enrollment were included. Regarding statin therapy, only patients who had not received any previous statin therapy or were off statin therapy for at least six months were included.

Patients with PDR, pregnant or lactating women, individuals with hepatic or renal insufficiency, those with evidence of systemic inflammation or malignancy, and those with elevated creatine phosphokinase or deranged liver function tests (LFTs) or kidney function tests (KFTs) were excluded from the study.

Baseline evaluation included the collection of demographic details and systemic risk factors (comorbidities). Demographic information included the patient’s age, duration of diabetes, associated systemic risk factors, and comorbidities such as hypertension (HTN), coronary artery disease (CAD), chronic kidney disease (CKD), and neuropathy.

Ocular examination included best corrected visual acuity (BCVA), intraocular pressure (IOP), slit lamp anterior segment evaluation, and posterior segment evaluation using 90D biomicroscopy.

All patients underwent baseline fundus photography (Visupac FF 450 plus, Carl Zeiss Meditech AG, Germany or DRI Triton, Topcon®, Japan), fundus fluorescein angiography (FFA) on confocal scanning laser ophthalmoscopy (CSLO) based fundus camera (Spectralis Heidelberg, Germany); and swept source OCT (DRI Triton, Topcon®, Japan) to measure central macular thickness (CMT).

The pupils of the patients were dilated by instilling 0.8% tropicamide. Forty-five-degree color fundus photographs were acquired using a digital fundus camera, with the images focused on the disc and macula. Swept source OCT (DRI Triton, Topcon®) 5-Line raster scan of the macula was done. The FFA was performed on a CSLO-based fundus camera (Spectralis Heidelberg). The scans were acquired multiple times, and the images with the least motion artefacts were selected for further analysis.

Baseline laboratory testing included Serum Vitamin D, PTH, calcium profile, fasting blood sugar (FBS), HBA1C, Lipid Profile, KFT, and LFT. FBS, HBA1C, Lipid Profile, KFT, and LFT were repeated every 3 months. Vitamin D loading was administered to all patients before initiating long-term statin therapy, in consultation with an endocrinologist. Oral vitamin D sachets (containing 1 lakh IU per sachet) were administered for the initial 10 days, followed by monthly administration.

The patients were randomized into two groups. Patients in group A were randomized to receive low-dose atorvastatin (10-20 mg), while patients in group B received high-dose atorvastatin (30-40 mg). Following this, the patients received three loading injections of ranibizumab (0.5 mg in 0.05 ml, injection Accentrix) at one-month intervals. Following this, the patients were treated according to a Pro re nata (PRN) regimen over six months.

The primary clinical outcome measure was the number of injections required at 6 months. The secondary clinical outcome measures were final best-corrected visual acuity (BCVA) and central macular thickness (CMT) at 6 months. The laboratory outcome measure included serum VEGF levels measured at baseline and at 6-month follow-up.

### VEGF analysis

One ml of peripheral blood sample was collected and stored at -80 degrees Celsius for VEGF analysis at baseline and 6 months. Serum VEGF was measured and quantified in the AEC lab. The total VEGF was measured in serum by using BD^TM^ CBA Human VEGF Flex Set, bead-based immunoassay catalog No. 558336, and BD CBA Human Soluble Protein Master Buffer Kit (Cat. No. 558264 or 558265). The procedure was followed as per the kit instructions. After sample acquisition on a flow cytometer (BD^TM^ LSR Fortessa), the results were generated using FCAP Array ^TM^ software in both graphical and tabular formats.

### Statistical analysis

The analysis was performed on the Statistical Package for Social Sciences (SPSS version 22, SPSS, Inc., Chicago, IL, USA). Descriptive statistics were used to analyze demographic data. The continuous variables were represented as means and standard deviations, and the categorical variables as percentages. The categorical variables between the two groups were compared using a chi-square test, and quantitative variables were compared using a paired t-test (baseline and follow-up) and an unpaired t-test to compare the changes between the two groups.

## Results

A total of 18 patients (12 males and six females) diagnosed with NPDR and CSME were included in the study. The mean age of the study population was 60.33 ± 8.65 years (range: 49-86 years). **Table 1** presents the baseline demographic profiles of patients in groups A and B.

**Table 1:** Baseline demographic profile of group A and group B

	Group A (n=9)	Group B (n=9)	p value
Mean age (years)	59.44 ± 11.80	61.22 ± 4.23	0.680^*^
Male: Female	2:1	2:1	1^^^
Associated systemic illness			
-HTN	5	8	0.114^**^
-CAD	1	2	0.527^**^
-CKD	1	2	0.52^**^
Duration of DM (years)	11.55 ± 6.12	11.66 ± 8.18	0.974^*^
Phakic: Pseudophakic	8:1	7:2	0.52^**^

*unpaired t test, **chi square test, ^ Fischer exact test HTN = Hypertension, CAD = coronary artery disease, DM = Diabetes mellitus, CKD = chronic kidney disease

The baseline metabolic profile (FBS, HbA1c, serum TG, serum LDL, serum cholesterol, serum HDL, SGOT, SGPT, Urea, and Creatinine) and baseline ocular parameters were comparable between the two groups (**Table 2**).

**Table 2:** Baseline metabolic and ocular parameters of group A and group B

Baseline metabolic pro			
	Group A	Group B	p value*
FBS (mg/dl)	157.23 ± 56.67	163.21 ± 58.70	0.829
HBA1c (%)	8.06 ± 1.63	8.76 ± 2.84	0.534
Serum TG (mg/dl)	136.41 ± 54.58	207.43 ± 87.23	0.058
Serum LDL (mg/dl)	74.38 ± 18.78	136.23 ± 102.27	0.109
Serum cholesterol (mg/dl)	158.70 ± 16.88	216.53 ± 104.67	0.139
Serum HDL (mg/dl)	54.87 ± 11.63	49.99 ± 11.63	0.348
SGOT (U/L)	26.55 ± 5.41	28 ± 5.56	0.584
SGPT (U/L)	31.66 ± 6.20	31.11 ± 4.62	0.832
Urea (mg/dl)	23.75 ± 11.81	24.42 ± 14.89	0.917
Creatinine (mg/dl)	0.91 ± 0.28	0.92 ± 0.23	0.929
**Baseline ocular parameters**			
BCVA (logMAR)	0.690 ± 0.387	0.615 ± 0.264	0.877
IOP (mmHg)	14.33 ± 4.18	13.55 ± 2.78	0.650
CMT (μm)	516.77 ± 139.19	438.77 ± 80.23	0.169

*Unpaired t-test FBS = Fasting blood sugar, TG = Triglycerides, LDL = Low density lipoproteins, HDL = High density lipoproteins, SGOT = Serum glutamic oxaloacetic transaminase, SGPT = Serum glutamic oxaloacetic transaminase

### Clinical outcomes

The primary clinical outcome measure was the total number of injections required during the study period in both groups. In our study of 18 patients, the mean number of injections administered was 3.4 ± 0.615. The total number of anti-VEGF injections required in groups A and B was 3.55 ± 0.72 and 3.33 ± 0.5, respectively. The difference between the two was not statistically significant (p-value: 0.24). Additionally, the number of injections required after three loading dosages (monthly) in group A (0.55 + 0.72) and group B (0.33 + 0.50) was also not statistically significant.

The secondary outcome measures included changes in BCVA and CMT at 6 months relative to baseline in both groups.

Intragroup analysis of group A (**Table 3**) revealed a significant improvement in BCVA at 3 months (0.299 ± 0.189 logMAR) and 6 months (0.270 ± 0.135 logMAR), respectively, compared to baseline (0.690 ± 0.387 logMAR). Similarly, CMT showed a significant reduction at 3 months (269.66 ± 85.57 μm) and 6 months (243.00 ± 30.30 μm) compared with baseline (516.77 ± 139.19 μm).

**Table 3:** Intragroup analysis of various ocular parameters in group A and group B

*Group A (anti-VEGF+ low-dose statin)*			
	**Baseline**	**3 months (10-14 weeks)**	**6 months (22-26 weeks)**
**BCVA (logMAR)**	0.690 ± 0.387	0.299 ± 0.189 (p value = 0.025)^*^	0.270 ± 0.135 (p value = 0.013)
**CMT (μm)**	516.77 ± 139.19	269.66 ± 85.57 (p value = 0.001)	243.00 + 30.30 (p value = 0.002)
*Group B (anti-VEGF + high dose statin)*			
**BCVA (logMAR)**	0.615 ± 0.264	0.535 ± 0.328 (p value = 0.05)	0.574 ± 0.305 (p value = 0.372)
**CMT (μm)**	438.77 ± 80.23	343.66 ± 90.27 (p value = 0.007)	362.62 ± 129.32 (p value = 0.136)

*Paired t-test for intragroup analysis

Intragroup analysis of group B (**Table 3**) showed significant improvement in BCVA at 3 months (0.535 ± 0.328 logMAR), but not at 6 months (0.574 ± 0.305 logMAR), compared to baseline (0.615 ± 0.264 logMAR). Similarly, CMT showed a marked improvement at 3 months (343.66 ± 90.27 μm) compared to baseline (438.77 ± 80.23 μm).

Intergroup analysis showed a significant improvement in BCVA and CMT at 6 months of follow-up in the low-dose statin group (Group A) as compared to the high-dose statin group (Group B) [p value=0.02 and p value=0.035, respectively] (**Table 4**).

**Table 4:** Intergroup analysis of ocular parameters between group A and group B

	Baseline			3 months (10-14 weeks)			6 months (22-26 weeks)		
	Group A	Group B	p value^*^	Group A	Group B	p value	Group A	Group B	p value
BCVA (logMAR)	0.690 ± 0.387	0.615 ± 0.264	**0.877**	0.299 ± 0.189	0.535 ± 0.328	**0.085**	0.270 ± 0.135	0.574 ± 0.305	**0.02**
CMT (μm)	516.77 ± 139.19	438.77± 80.23	**0.169**	269.66 ± 85.57	343.66 ± 90.27	**0.093**	243.00± 30.30	362.62 ± 129.32	**0.035**

### Laboratory outcomes

The baseline serum VEGF level in our study population (n = 18) was 70.92 ± 49.38 pg/ml. The mean baseline serum VEGF levels in groups A and B were 63.12 ± 36.57 pg/ml and 78.68 ± 60.90 pg/ml, respectively, and the difference was not statistically significant (p value = 0.523).

The serum VEGF levels decreased at the 6-month follow-up (46.59 ± 8.16 pg/ml; p = 0.375) in group A, although the decrease was not statistically significant. However, the values in group B increased (91.04 ± 96.36 pg/ml; p value = 0.443) as compared to baseline.

Intergroup analysis at 6 months showed no difference in serum VEGF levels between groups A and B (p = 0.205).

No correlation was observed between serum VEGF and CMT at baseline and 6 months overall, as well as individually in groups A and B (p-value> 0.05) (**Table 5**).

**Table 5:** Correlation of serum VEGF with CMT at baseline and 6 months in group A and group B

	Pearson correlation coefficient (Baseline)^*^	Pearson correlation coefficient (6 months) ^*^
Overall	-0.107	-0.35
Group A	0.205	0.066
Group B	-0.344	-0.309

*Pearson correlation test

## Discussion

Statins are an essential class of lipid-lowering drugs that inhibit HMG-CoA reductase, an enzyme involved in the synthesis of mevalonate and cholesterol [**26**]. In addition to its lipid-lowering action, several non-lipid-lowering effects have been described in the literature [**27**]. Studies have shown vasculoprotective effects with statin therapy, thus reducing the incidence of cardiovascular mortality and morbidity and cerebrovascular accidents like stroke, especially in diabetic patients [**28,29**]. The drug is widely used for its systemic benefits, and its effects on the retina have been demonstrated by resolving hard exudates in CSME [**13**].

This prospective randomized interventional study was conducted to assess the effectiveness of statin therapy in the management of type 2 diabetic patients with NPDR with CSME. We found that statins, administered at a dose of 10-20 mg daily, were effective as an adjunct to standard-of-care anti-VEGF therapy in CSME, providing better functional and structural outcomes at 6 months of follow-up.

Experimental studies have shown conflicting results of statin therapy on angiogenesis. Although Kureishi et al. [**30**] have demonstrated that statins promote angiogenesis, an in vitro study by Vincent et al. [**31**] has shown that statins inhibit angiogenesis. In addition, studies have shown a dose-dependent, lipid-independent biphasic effect of statins on angiogenesis, with low-dose statins exhibiting pro-angiogenic activity and high-dose statins exhibiting anti-angiogenic activity [**17,18**]. In contrast, other experimental studies on mouse models have demonstrated the beneficial effects of low-dose statin therapy on ischemic retinopathies and choroidal neovascularization [**21,32**]. Thus, studies evaluating the dose-dependent effects of statins are typically conducted in vitro or using animal models, and similar clinical studies assessing the dose-dependent action of statins on angiogenesis are limited [**22**].

Dworacka et al. have demonstrated that low-dose statins (atorvastatin 10 mg and simvastatin 10-20 mg) can significantly reduce serum VEGF levels in diabetic patients [**19**]. Similarly, Giurgea et al. have demonstrated a significant reduction in serum VEGF concentration after simvastatin therapy (20 mg and 40 mg), with a similar decrease in VEGF observed in both dosing groups [**22**]. This highlights that statins have an anti-angiogenic effect, but whether this effect is dose-dependent remains a matter of debate. Hence, we conducted this pilot study to also evaluate the anti-angiogenic effect of statins at different dosing levels, specifically low dose (10-20 mg) versus high dose (30-40 mg).

Not only are statins associated with a reduction of serum VEGF, but they also tend to reduce local ocular VEGF concentration [**20**]. Simvastatin has been shown to effectively lower the intravitreal concentrations of permeability and angiogenic factors, such as VEGF and angiopoietin 2, in both diabetic and non-diabetic patients. In their experimental studies on rats, Ekerbicer et al. have shown that statins reduce ocular VEGF levels in both diabetic and non-diabetic rats [**20**]. Not only did statins reduce VEGF levels, but they were also found helpful in eyes with preexisting retinopathy.

Our study showed that the mean number of injections required over 6 months was comparable between the two groups (10-20 mg versus 30-40 mg). This could be attributed to a shorter follow-up period and warrants similar longitudinal studies with longer follow-up.

In our study of 18 patients, the mean number of injections administered was 3.4 ± 0.615, which is less than what has been reported in real-world experience. Studies on real-world experience with anti-VEGFs in DME required a mean of 5 ranibizumab injections at the end of 6 months [**33**]. We believe that the lower number of injections required in our study group could be attributed to local anti-VEGF action associated with statin therapy. However, we did not measure the vitreous VEGF levels in our study.

Regarding the dose-dependent effect of statins, we did not find any difference in the number of anti-VEGF injections between the two groups. However, low-dose statins were associated with better visual acuity and CMT at 6 months of follow-up compared with the high-dose statin group, indicating better structural and functional outcomes in the low-dose statin group. This could be attributed to the fact that statins, apart from their lipid-lowering action, have several lipid-independent pleiotropic effects, including improving or restoring endothelial function, decreasing oxidative stress, and vascular inflammation, as well as antithrombotic and immunomodulatory activity, which can lead to improvements in diabetic retinopathy and diabetic macular edema [**21**].

The baseline serum VEGF level in our study population (n = 18) was 70.92 ± 49.38 pg/ml. A recent meta-analysis has shown that serum VEGF levels in diabetic patients range widely, from 92.29 ± 27.90 pg/ml to 775.13 ± 770.20 pg/ml [**34**]. Diabetic patients tend to have higher serum VEGF levels compared to non-diabetic patients [**35**]. Additionally, serum VEGF levels exhibit a positive correlation with the stage of the disease, with proliferative diabetic retinopathy (DR) having significantly higher values than the non-proliferative stage of the disease [**36**]. Metabolic profiles, including HbA1c values, also correlate with serum VEGF levels. We believe that the lower baseline serum VEGF levels in our patients, compared with those reported in previous studies, are primarily due to the inclusion of patients with NPDR and reasonable metabolic control.

Our study showed no correlation between serum VEGF and CMT both at baseline and 6 months. Our results are consistent with those of previous studies, which found no correlation between serum VEGF and CMT [**37**]. A lack of correlation between serum VEGF and CMT indicates the importance of local VEGF production in DME. Secondly, other systemic comorbidities and microvascular complications can affect serum VEGF levels [**38,39**]. Therefore, the lack of correlation between serum VEGF and CMT can be explained by the multifactorial dependence of serum VEGF levels.

Lastly, low-dose statins showed a trend toward a reduction in serum VEGF over 6 months, whereas high-dose therapy showed a rising trend. Although the values did not reach statistical significance, a longer follow-up is required to determine whether any effect exists. Although these values reflect the underlying systemic VEGF levels in these patients, which are critical for VEGF analysis, the role of blood sample collection, preservation, and processing should also be considered.

A declining trend in serum VEGF in group A, compared to a rising trend in group B, suggests that low-dose statin therapy has an anti-VEGF effect. This finding contrasts with previous reports of pro-angiogenic action at low doses and anti-angiogenic action at high doses of statins, which require further investigation in larger clinical trials [**17,18**]. This is probably because the studies mentioned above were experimental and did not include subjects with diabetes. It is believed that statin activity is significantly modified by underlying diabetes [**19**].

Our study has a few limitations. Firstly, the follow-up period was limited to 6 months. Secondly, although this was a prospective study, patients were unable to follow the scheduled visit protocol due to pandemic-related logistical constraints. Our country was severely impacted by the 1st and 2nd waves of COVID-19, which overlapped with the study period. Thirdly, we did not analyze vitreous VEGF levels due to the inherent limitations of the invasive intraocular procedure. Lastly, the patients underwent systemic investigations at various laboratories, as deemed suitable and convenient.

## Conclusion

To summarize, we found that the number of anti-VEGF injections decreased, with oral statins administered as an adjunct in patients with CSME. Additionally, statins were significantly effective in improving BCVA and reducing CMT when administered at a standard dose (10-20 mg). We believe that statins may play a role in clinical practice as an adjunct to anti-VEGF therapy in all patients with CSME, leading to better functional and anatomical recovery.
